# The Use of Natural Methods to Control Foodborne Biofilms

**DOI:** 10.3390/pathogens12010045

**Published:** 2022-12-27

**Authors:** Michelle Marie Esposito, Sara Turku

**Affiliations:** 1 Department of Biology, College of Staten Island, City University of New York, 2800 Victory Blvd, Staten Island, New York, NY 10314, USA; 2 PhD Program in Biology, The Graduate Center, City University of New York, New York, NY 10016, USA; 3 Macaulay Honors College, City University of New York, New York, NY 10023, USA

**Keywords:** biofilms, bacteriocins, bacteriophages, phytochemicals, synergy, antimicrobials

## Abstract

Biofilms are large aggregates of various species of bacteria or other microorganisms tightly attached to surfaces through an intricate extracellular matrix. These complex microbial communities present quite the challenge in the food processing industry, as conditions such as raw meats and diverse food product content in contact with workers, drains, machinery, and ventilation systems, make for prime circumstances for contamination. Adding to the challenge is the highly resistant nature of these biofilm growths and the need to keep in mind that any antimicrobials utilized in these situations risk health implications with human consumption of the products that are being processed in these locations. For that reason, the ideal means of sanitizing areas of foodborne biofilms would be natural means. Herein, we review a series of innovative natural methods of targeting foodborne biofilms, including bacteriocins, bacteriophages, fungi, phytochemicals, plant extracts, essential oils, gaseous and aqueous control, photocatalysis, enzymatic treatments, and ultrasound mechanisms.

## 1. Introduction

As worldwide economies and inhabitants have become more and more dependent on manufactured foods, it is important to trust in these institutions that they are keeping up with proper sanitary protocols. Many of these factories risk the contamination of food through the processing of raw materials, as well as the presence of workers, drains, and ventilation systems [1]. Foodborne biofilms are of particular concern in the food processing industry, with *Listeria monocytogenes*, *Bacillus cereus*, *Escherichia coli O157:H7*, *Salmonella* spp., *Pseudomonas* spp., and *Staphylococcus aureus* all found to be particularly adherent strong formers of biofilms on foods and food preparation surfaces [1]. Biofilm development microorganisms can be classified as pathogenic (*B. cereus*, *E. coli*, *L. monocytogenes*, and *Salmonella enterica Enteritidis* and *Typhimurium* serotypes) and/or as spoilage microbes (*B. cereus* and *P. aeruginosa*) [2]. While initial discoveries of microorganisms focused on planktonic, meaning free or singular, forms of existence, the ability of different species of microorganisms to aggregate into groups of self-producing matrices called biofilms has presented unique challenges to various aspects of everyday human life [3]. Biofilm formation by these organisms occurs in approximately five stages, which includes initial reversible landing or attachment onto a surface, irreversible aggregation via electrostatic forces, microcolony formation secreting extracellular polymers, growth and maturation including quorum sensing molecules, and lastly dispersion or detachment due to disruptive forces [3].

**Figure 1:**
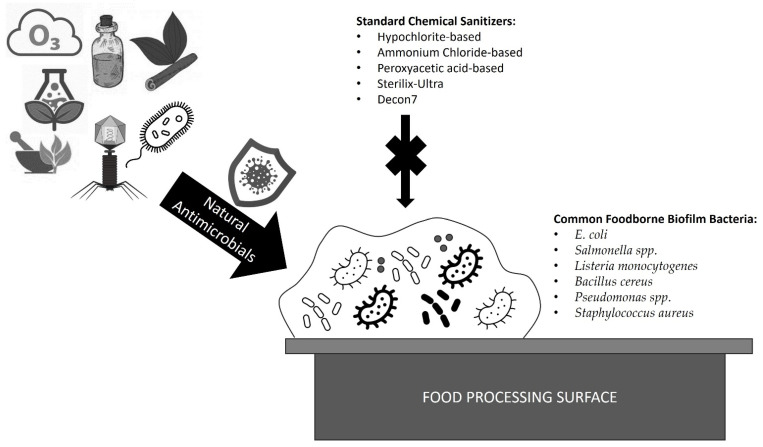
Natural alternatives as antimicrobials against common foodborne biofilms. Bacteriocins, bacteriophages, fungi, phytochemicals, plant extracts, essential oils, gaseous and aqueous control, photocatalysis, enzymatic treatments, and ultrasound mechanisms demonstrate promising antimicrobial activities as alternatives to harsh toxic chemicals against common foodborne biofilm pathogens, including species of *Escherichia*, *Salmonella*, *Listeria*, *Bacillus*, *Pseudomonas*, and *Staphylococcus*.

When targeting foodborne biofilms, many standard sanitizers are presented with the problem that, while some residents of the biofilm are greatly sensitive, others remain far less sensitive and protected by intricate organic polymer matrices [1,4]. For instance, one study demonstrated that *L. monocytogenes* 99-38 was most sensitive to standard sanitizers (such as hypochlorite-based sanitizers, ammonium-chloride-based sanitizers, peroxyacetic-acid-based sanitizers, as well as newer Sterilex-Ultra and Decon7) whereas *E. coli* F4546 and *S. Montevideo FSIS 051* were far less sensitive to all treatments [1]. This was attributed to the *E. coli* and *Salmonella*’s ability to produce more extensive extracellular polymeric substances (EPS) characteristic of biofilm formations [1]. EPS are organic matrices that enhance the cohesion of complex biofilm structures [4]. Residing in mixed-species biofilms provides added layers of protections for foodborne pathogens, increasing resistance to biocides, and reducing the effectiveness of antimicrobial techniques [5]. As many biocides are developed via testing on pure cultures, it is important to consider the effect that complex biofilm formation can have on the usefulness of these treatments in robust mixed cultures and to consider more effective means of targeting these problematic microbial communities [5]. In this review, natural means of targeting and eradicating foodborne biofilms will be explored, including the use of bacteriocins, bacteriophages, fungi, phytochemicals, plant extracts, essential oils, gaseous and aqueous control, photocatalysis, enzymatic treatments, and ultrasound mechanisms (Figure 1). Not only can natural compounds serve to reduce growths as cleaning compounds, but due to their natural origins can also serve as potentially safe additives to food products to reduce spoilage and prevent food contamination [6].

## 2. Bacteria, Viruses, and Fungi to Control Biofilms

Newly identified strains of lactic acid bacteria (LAB) are one potential option for the natural control of biofilms that are usually characterized by strong antimicrobial resistance and the potential for foodborne disease spread [6]. One study identified six new high-acidification strains with significant antagonistic properties capable of repressing embedded pathogen biofilms of *S. aureus*, *E. coli*, and even *Pseudomonas aeruginosa* (although to a lesser extent than others) at levels more significant than current industrial probiotic *L. plantarum 8PA_3_* [6]. The six strains were named *Lactobacillus plantarum* (AG1, AG9, AG10, and AG15) and *Lactobacillus fermentum* (AG8, AG16) [6]. More importantly, not only did the six strains exhibit potent abilities to eradicate pathogen biofilms, they also demonstrated strong tolerance to the acid and bile gastric conditions simulated (especially strain AG10), which is quite promising for use as additives or probiotics [6]. The antimicrobial strains also demonstrated additional potential in the food industry with the ability to ferment milk and exhibited valued properties of the most currently used yogurt strains, *L. bulgaricus* and *S. thermophilus*, including protein content [6]. Taken all together, these six strains have been considered a promising option to reduce dangerous chemical biocins and improve the development of environmentally sound tools against foodborne biofilms in the food processing industry [6].

Another bacteriocin that has shown to be promising in handling foodborne biofilms is the DF01 bacteriocin isolated from Lactobacillus brevis [7]. Through microtiter plates, fluorescent microscopy, and scanning electron microscopy, DF01 bacteriocin has been demonstrated to successfully diminish the formation of foodborne biofilms of *E. coli* and *S. typhimurium*, although it failed to effectively remove the already established biofilms of those strains [7]. Ultimately, this bacteriocin is still valuable as the preventative nature of its biofilm-targeting abilities did reduce these foodborne biofilms of stainless steel surfaces as it controlled formation steps [7]. Unlike some bacteriocins, the D01 bacteriocin, which was isolated from the Korean fermented vegetable Dongchimi, exhibits some limitation in use, as it is sensitive to α-amylase and proteolytic enzymes due to its categorization as a class IV bacteriocin, meaning it contains glycoprotein, as opposed to some bacteriocins, such as class I bacteriocin nisin A [7,8]. Whereas DF01 bacteriocin was isolated through the study of fermented vegetables, lactic acid bacteria (LAB) from fermented fish and fermented chicken have also proven to be valuable [9]. Interestingly, those lactic acid bacteria from the fish and chicken develop into biofilms that have been shown to have potential against pathogenic biofilms [9]. In other words, LAB biofilms can fight foodborne biofilms, which include biofilms composed of B. cereus ATCC 11778, *E. coli* ATCC 8739, and Salmonella enterica subsp. enterica serovar Typhimurium ATCC 13,311 [9]. These LAB biofilms successfully showed the ability to fight planktonic and biofilm forms of foodborne pathogens and the ability to prevent biofilm formation steps through a competitive mechanism [9]. Furthermore, one of the benefits of utilizing bacteriocins and naturally occurring compounds from microorganisms is that it opens a vast reservoir of potential antimicrobials due to the endless discoveries of novel microbes and microbial compounds. For instance, bacteriocin BaCf3 isolated from *Bacillus amyloliquefaciens* BTSS3 was confirmed through sequencing to be novel and to contain disulfide linkages that make it a robust and stable compound [10]. In microarray inhibitory concentration experiments, BaCF3 was shown to decrease biofilms by up to 80% when used at even low concentrations against strong foodborne biofilm producers, such as *S. typhimurium*, *C. perfringens*, and *E. faecalis* [10]. Inhibition of biofilm formation was even more robust, with percentages of preventative effectiveness being up to 100% in the case of *S. typhimuium* and *E. faecalis* [10]. BaCf3 even inhibited foodborne biofilms of *P. aeruginosa* and *B. casei*, both of which are known for their high resistance to many common antibiotics or antimicrobial treatments [10]. Most importantly, cytotoxicity assays with mouse- and rat-derived cell lines demonstrate that BaCF3 does not inhibit animal cells, which is a promising result for the use of this bacteriocin in food preservation [10]. Furthermore, efficacy can be enhanced via the use of nanoparticle vesicles that allow for extended release, improved bioavailability, and improved binding to bacterial surfaces [11]. For instance, the development of rhamnolipid rhamnosome nano-vesicles loaded with nisin bacteriocin increased the encapsulation efficiency of the bacteriocin up to 88% from just 47%, and observed biofilm mass reduction of mixed Gram-positive and Gram-negative foodborne species was approximately 80% [11].

It has also been shown that the use of essential oils, such as *Origanum vulgare*, *Cinnamomum cassia*, *Brassica hirta*, *Thymus vulgaris*, *Satureja montagna*, and *Cymbopogon nardus*), can have synergistic effects in combination with bacteriocins, such as nisin, pediocin, and extracts from *Enterococcus faecium* MT 104 and MT 162 [12]. These essential oils, particularly in combination with nisin, pediosin, and MT isolates, showed antimicrobial effects against foodborne pathogenic strains, *B. cereus*, *E. coli*, *L. monocytogenes*, *S. typhimurium* and *S. aureus*, and food spoilage strains (*Lactobacillus sakei* and *Pseudomonas putida*). This natural alternative to harsh synthetic additives could potentially improve the bacteriocin abilities being observed with new LAB strains as well. Essential oils, which will be discussed later in this review, have many promising antimicrobial abilities but are limited in their usefulness, especially in the food industry, due to the fact that the higher concentrations of essential oils necessary for antimicrobial food preservation can yield unwanted side effects, such as organoleptic (smell, taste, and texture) food quality changes [12]. By finding synergistic or additive effects with these essential oils and bacteriocins, lower concentrations would be able to be utilized to minimize unwanted side effects and serve as a means to overcome the food industry’s dependence on questionable synthetic additives [12].

In addition to bacterial means of controlling biofilms, it should be noted that viruses have the potential to be just as valuable as well. *E. coli* is one of the most persistent foodborne biofilm inhabitants that pesters the food industry [13]. In one study targeting biofilms of various strains of *E. coli* grown on stainless steel, rubber, or lettuce, analyzed via susceptibility testing, field emission scanning electron microscopy, and confocal laser scanning microscopy demonstrated that bacteriophage BPECO 19 was able to significantly reduce the amount of adhered biofilm cells [13]. In comparison to other natural means, such as the phytochemical plant extract methods, it should be noted that the use of bacteriophages requires extra screening or very selective decisions with regards to strains, as these viruses could potentially disrupt microbiomes or present challenges of emergence of resistance, lysogenic transformations, pathogenicity gene transmission, or endotoxic effects upon the destruction of Gram-negative bacteria [14]. Furthermore, some concerns exist regarding the lack of knowledge currently available surrounding any potential phage-mediated ecological perturbations that could occur [14,15]. One technical challenge of phage use in food products that has been an obstacle is that, while initial decreases in contaminating bacteria occur, a lack of subsequent elimination is not observed [16,17,18]. This lack of expected lytic exponential activity of phages in food products is hypothesized in one study to be due to the inability of progeny phages to move successfully through food matrices, especially in foods that lack high moisture levels, and thus the progeny phages fail to then encounter substantial subsequent bacteria beyond the initial antimicrobial activity [16]. Additionally, as with many antimicrobial methods, the emergence of phage-resistant bacteria is a concern, and would require intervention techniques, such as phage cocktails and the consistent re-screening of potential phage strains [16]. Although theoretical concerns exist, the strict screening of strains helps minimize these concerns and allows for great specificity that has even been shown to prevent any impacts on the host’s microbiome [19]. In addition to technical concerns, it should be noted that another major issue in the use of phages in food products is consumer acceptance [16]. The public would need thorough awareness training as it can be very concerning to hear that food has been sprayed or modified to include viruses without an understanding of the actual safety of such products [16].

When the correct bacteriophage is selected, it can be a powerful resource, as it can potentially have great host specificity and stability and could be produced as phage cocktails to maximize the targeted effects on mixed-species aggregates of biofilms [13]. This has also proven to be a successful technique against *Salmonella*, a common contaminant of food products, resident of many food-processing-facility biofilms, and was credited with over USD 2.5 million of revenue loss in the US in 2007 [20]. Cocktails of broad lytic phages from the families *Siphoviridae*, *Ackermannviridae*, and *Podoviridae* have been shown to reduce a variety of *Salmonella* serotypes in milk, chicken breasts, and biofilms on microplates, as well as stainless steel surfaces at 4 °C and 25 °C [20]. The selected phages were able to be procured from a collection of 42 phages that had been isolated from environmental water samples and narrowed down via spot testing and lytic activity testing to determine the three most broad lytic phages against the most diverse amount of *Salmonella* serotypes [20]. With more than 10^31^ phages estimated worldwide [21], this process could have very potent potential in the targeted control of biofilms. Whereas the bacteriophage studies described here so far analyzed *E. coli* biofilms and *Salmonella* biofilms separately, a recent study combined these two prominent foodborne biofilm contaminants to analyze phage effectiveness in these even more diverse conditions [22]. The novel polyvalent phage STP55 of the family *Ackermannviridae* has been discovered to be capable of lysing *Salmonella* and *E. coli* serotypes at acid and basic pH values ranging from 4 to 12, as well as a wide range of temperatures from 30 to 60 °C [22]. This effectiveness included dual-species biofilms grown on lettuce [22]. Overall, STP55 has been shown to be an efficient antimicrobial capable of inhibiting single- and dual-species biofilm formation, as well as capable of biofilm removal, all at a promising range of pH and temperature values commonly found in food preparation [22]. With more and more antibiotic-resistant bacterial strains emerging in biofilms, bacteriophages could be a powerful means to target the more persistent species while appearing to be safe themselves [22]. Genomic analyses demonstrated that STP55 has a short latent period and large burst size for hearty antimicrobial activity but lacks any antibiotic-resistance genes, virulence factor genes, or integrases that would permit a phage to integrate or harm the host genome [22,23]. Furthermore, phages are considered to be very consumer-friendly as they meet the qualifications to be labeled organic, kosher, or halal, thus expanding their versatility or reach in the food market [24].

Whereas *E. coli* and *Salmonella* tend to be foodborne pathogens that get the most attention with the common public, *B. cereus* is another highly resistant widespread foodborne pathogen known to produce biofilms and to plague the food industry [25]. As already mentioned, phages can be a uniquely ideal means of naturally treating or preventing the most resistant of biofilm bacteria. In one recent study, phages proved to be highly versatile against *B. cereus*, wherein 62 phages were isolated from sewage samples, and seven were found to have broad-spectrum lytic antimicrobial activity in the prevention and reduction of 174 *B. cereus* isolates [25]. Transmission electron microscopy demonstrated the phages to be *Siphoviridae*, and SDS-PAGE analysis of structural proteins, along with restriction analysis of genomes, showed strong relatedness but great diversity amongst the phages [25]. Most significant was the low-percentage-of-lysis characterization of these phages, as that was selected to reduce the chances of resistance development and minimize the potential harm to important normal flora [25]. Overall, however, bacteriophage studies point out that the success of bacteriophage treatment of biofilms is highly variable depending on factors, such as strains, temperature, multiplicity of infection (MOI), biofilm content or age, and the surfaces or foods involved [26]. For instance, a study using bacteriophage cocktails (LMPC01+02+03) to target and destroy *L. monocytogenes* foodborne biofilms demonstrated that, with these particular phages, biofilm maturity had a greater impact on the antibiofilm activities than temperature or surface material, whereas with other phage and biofilms, different factors can become the most significant [26]. Despite the variability and potential limiting factors, bacteriophage therapy is ultimately a very valuable tool in microbial control [27]. Current methods of targeting foodborne or enteric pathogens has relied heavily upon harsh chemicals or antibiotics, which lead to major microbiome/normal flora dysbiosis in humans that can in turn have drastic consequences, including periodontitis, cardiovascular diseases, neurodegenerative diseases, diabetes, more significant complications with infections, and even the development of cancers [27,28,29,30]. Therapeutic bacteriophage treatment has been shown to be capable of killing targeted common enteric pathogens, such as enteroaggregative *E. coli*, without significantly altering the important normal flora of the digestive tract [27]. In contrast, antibiotics, such as ciproflaxin, have been shown to decrease the diversity of the normal flora up to 40% and, in comparison to the phage treatments, destroyed normal flora so significantly that no DNA was able to be obtained for 16S analysis in the ciproflaxin samples [27].

Foodborne biofilms can be very difficult to treat, but one way of combatting this issue is the use of fungi, specifically mushroom extracts [31]. The exact mechanism behind this is unknown, but it is thought to be because of the effect on cell surface charge and cell wall changes [31]. One type of mushroom that was researched was the *Pleurotus flabellus* strain Mynuk and the effects of its polysaccharide extracts [31]. A variety of types of extracts were used from hot water extracts to methanol extracts. While most of them displayed antiadhesion activity, the hot water extract had an adhesion prevention greater than 50% when treating the clinical strain of *Enterococcus faecalis* [31]. This is likely because crude extracts tend to be more effective [31]. The antiadhesion activity, however, did change between the clinical strain and its American Type Culture Collection (ATCC) version [31]. The ability of the extracts to inhibit biofilm activity was comparable to the anti-adhesion activity but not as effective [31]. The antibiofilm activity also was the highest when used against clinical strains of Gram-positive bacteria but had a decreased effect for the ATCC versions again [31]. Overall, the extracts from the *P. flabellatus* strain Mynuk were shown to be more productive as antiadhesion agents than antibiofilm agents [31]. Extracts of the Basidiomycete *Grifola frondosa* mushroom also displayed the potential to treat biofilms [32]. Partially purified polysaccharides and hot alkali extract of that mushroom were observed to have a bactericidal effect towards *B. Cereus* [32]. The extract of the albino *G. frondosa* has also been shown to treat biofilms, specifically methicillin-resistant *S. aureus* [33]. It led to a significant reduction of optical density of the biofilm and category reduction [33]. Additionally, wild basidiomycete mushrooms and oyster mushroom extracts exhibited antiadhesion effects on foodborne biofilms [34,35]. On *E. coli*, the extracts caused adhesion inhibition percentages approximately between 26 to 49% and for *Listeria innocua*, it was approximately between 32 to 45% [34].

Peptides extracted from fungi have also been observed to treat biofilms [36]. An extracellular thermostable peptide found in *Aspergillus fumigatus* BTMF99, known as MFAP99, had 99% biofilm removal activity when used to treat *Bacillus pumilus* and led to the demolition of biofilm architecture [36]. The benefits of this method include the peptide functionality at a wide pH range and at high temperatures, as well as nontoxicity to human red blood cells [36]. An alternative treatment method using fungi is synthesizing nanoparticles from them [37]. Fungi are able to mass produce biosynthesized nanoparticles that do not need to perforate the cell as they can cause an effect via direct contact [37]. Silver nanoparticles synthesized from *Penicillium polonicum* demonstrated antibacterial effects, and, when used against *Acinetobacter baumanii*, the nanoparticles had a minimum inhibitory concentration of 15.62 μgmL^−1^ and a minimum bactericidal concentration of 31.24 μgmL^−1^ [37]. Additionally, after 6 h, they completely killed the bacterial cells in a killing kinetic assay [37]. 

## 3. Phytochemicals and Essential Oils 

Although bacteriophages, bacteriocins, and fungi have proven to be very promising means of antimicrobial activities, they are theoretically not without risk. Their microbial nature means could in theory cause harm to a human host under the right circumstances, especially if they lead to microbiome dysbiosis or have limitations due to variability concerns. Thus, it is important to explore other natural means of the antimicrobial control of foodborne biofilms as well, including the use of phytochemicals, plant extracts, and essential oils, which have the potential to also add value to food products while decreasing pathogen contaminations [38].

A major aspect of biofilm formation involves bacteria being able to work in collaboration with each other bacteria via cell-to-cell communication known as quorum sensing (QS) [39]. QS is a form of bacterial gene regulation in which expression is dependent upon cell-population density through a series of extracellular signaling molecules in cascades [39]. QS has been found to heavily influence biofilm formation in bacteria, as quorum-sensing cascades include genes involved in the production of exopolysaccharides and interspecies communication [39]. For instance, *Vibrio harveyi* has been shown to synthesize homoserine lactone (HSL) signal (HAI-1) as an intraspecies communication signal and furanosyl borate diester autoinducer (AI-2) as an interspecies communication signal, both of which promote quorum-sensing activities that can lead to biofilm formation [39]. Compounds that reduce or prevent QS abilities in bacteria can be very promising in controlling biofilms. One such compound identified is *Amomum tsaoko* (Zingiberaceae), which also goes by the names of “Black Cardamom” and “Tsaoko Amomum” across parts of Asia [38]. Extracts from this plant are already safely used in the treatment of ailments, such as gastrointestinal distress, malaria, and throat infections, and has been involved in various studies for its valuable anti-cancer, anti-inflammatory, and anti-microbial abilities among various other potentially beneficial traits [38]. Via MIC, anti-biofilm assays, and confocal laser scanning microscopy observation of biofilms and swarming motility testing, it has been determined that *A. tsaoko* extract is capable of inhibiting biofilm formation in a dose-dependent manner [38]. The *A. tsaoko* extract successfully demonstrated bactericidal activity, as well as reduced flagella-related activities, against various foodborne biofilm strains, including *S. aureus*, *S. Typhimurium*, and *P. aeruginosa* [38]. Although the results of such experiments with *A. tsaoko* are very promising, the exact mechanisms of action still remain elusive and further molecular testing must be done [38]. Initial mass spectrometry analyses have shown that *A. tsaoko* extract contains multiple chemical compound components already known to have antimicrobial activity [38]. One compound highlighted by the authors for its potential contribution to *A. tsaoko* extract activity is tsaokoaryline—a cytotoxic diarylheptanoid [7-(4-hydroxyl-3-methoxyphenyl)-1-(4 hydroxyphenyl)-hepta-4E,6E-dien-3-one]), which may impact the gene cascades of quorum-sensing regulation [38]. 

Another plant extract that has shown promising results in reducing quorum-sensing and virulence factors to inhibit biofilm formation is from *Laurus nobilis*, known in cooking as bay laurel, which is a type of evergreen tree [40]. Of greatest significance to targeting foodborne pathogens safely is that the laurel even showed effectiveness in inhibiting multidrug-resistant *S. aureus* strains, although for resistant strains, the effectiveness was more observable in planktonic specimens rather than the heavily protected biofilms [40]. When not dealing with multidrug-resistant species, then the foodborne biofilm formation prevention and antimicrobial activities were shown to be very potent and broad spread across both Gram-positive and Gram-negative bacteria [40]. This potent activity included the inhibition of biofilm formation and swarming motility [40], as was seen in the cardamom study as well [38]. Pyocyanin production was also reduced, which, like swarming motility, is an indication of virulence activity [40]. Since bay laurel is also known to have potent antioxidant properties, not only would it be of value as an antimicrobial additive, but it could also be a natural means to increase the health value of the food products that it is used in as a preservative or supplement [41,42,43]. As with bay laurel, other culinary plant products, such as garlic, onion, and cinnamon, have also been tested for anti-biofilm effectiveness as natural alternatives in food processing facilities [44]. Through disk diffusion, minimum inhibitory concentration, and crystal violet assays, it was shown that these three commonly used food compounds were able to inhibit initial cell attachment, as well as to a lesser degree, six hour preformed biofilms of foodborne pathogen *L. monocytogenes* [44]. 

It appears the most reactive antimicrobial compounds within these plant products were sulfides in onion and garlic extracts, and cinnamaldehyde in the cinnamon extracts [44]. Also found in leeks and clove, for instance, sulfides in garlic have been shown to have wide-spectrum antimicrobial activities and would thus work well against many other common strains of foodborne pathogens, including *S. aureus*, *E. coli*, *Vibrio*, *Y. enterocolitica*, and *H.pylori* [45,46]. Cinnamaldehyde is also known for wide-spectrum abilities against foodborne pathogens, such as *E. coli*, *S. aureus*, *S. typhimurium*, *B. cereus*, and *Y. enterocolitica*, with synergistic abilities apparent when in combination with carvacrol [47,48,49]. Some studies pinpoint the antimicrobial mode of action of cinnamon oil, cinnamaldehyde, and carvacrol, as well as other natural antimicrobials, as causing a leak of phosphate ions and decreasing intracellular adenosine triphosphate (ATP) [50]. Additionally, these compounds have been associated with modifications of phospholipid packing mechanisms and thus the disruption of bacterial cell membranes [50]. In keeping with a culinary perspective, oregano has also been shown to have a similar antimicrobial mechanism of reducing ATP and bacterial membrane structures, which makes sense, as carvacrol is a phenolic monoterpenoid found in the essential oils of oregano, thyme, pepperweed, and wild bergamot [50,51]. Due to that phenolic component, oregano oil was one of the early natural compounds studied to evaluate the ability of plant oils to reduce biofilm formation [52]. It was shown to effectively inhibit foodborne biofilms of *S. aureus* and *S. epidermidis*, as well as inhibiting the planktonic forms of these strains [52]. Since carvacrol oils of oregano are also known for additional anti-inflammatory, anti-oxidant, anti-cancer, and hepatoprotective properties, it further increases the value in the food industry [53,54,55]. It should be noted that, since thyme also contains carvacrol, it has also been identified as a valuable essential oil, as well as tea tree oil from *Melaleuca alternifolia*, against foodborne biofilms [56]. Thyme, due to its high thymol content, is already used for cleaning surfaces and removing dirt without any of the harmful and noxious odors that chemical cleaning products contain [56]. In one study, thyme and tea tree oil were shown to be very effective against *E. coli O157:H7*, *L. monocytogenes*, and *Salmonella* species on abiotic surfaces, including stainless steel, rubber, and minimum biofilm eradication concentration (MBEC) surfaces, with a reduction of biofilm cells by as much as 3.5 log CFU/cm^2^ [56]. 

Although many people think of the potent or harsh chemicals used in aromatherapy when they hear the term essential oils; this term simply just means oils extracted from plants, such as thyme, cinnamon, fennel, oregano, or mint [57]. Essential oils, such as thyme and tea tree oil, are very promising in the future of foodborne biofilm prevention and reduction, especially when multidrug-resistant pathogens are involved. Essential oils extracted from *Cymbopogon flexuosus* and *Thymus vulgaris*, for instance, have been demonstrated to result in strong inhibitory zones of multidrug-resistant *Enterococcus* species and *Aeromonas* species [58]. It should be noted, however, that even though these two essential oils had very promising results on planktonic cells and on *Aeromonas* biofilms, they failed to remove enterococcal biofilms [58]. Although that may be seen as a failure of the goal, it is not indicative of a failure of essential oils in the overall purpose of fighting foodborne biofilms, since as mentioned earlier, synergistic effects with the right pairing of essential oils could overcome instances wherein individual essential oils lack full antimicrobial efficacy [59]. Synergy of essential oils could provide great versatility and broaden the spectrum of effectiveness. Not only is the already described synergy between individual essential oils valuable, but studies have shown that essential oils can also be used in conjunction with antibiotics to yield synergistically improved effectiveness while preventing the spread of antibiotic resistance [60]. For instance, essential oil compounds thymol and cinnamaldehyde have been shown to produce synergistic effects with streptomycin when targeting *L. monocytogenes*, while cinnamaldehyde or eugenol produce synergy with streptomycin when used against *S. typhimurium* [60]. Cinnamaldehyde and citric acid are not just valuable in conjunction with antibiotics or other essential oils but also have proven valuable in conjunction with bacteriocins, such as the *Lactococcus lactis*-produced toxin called nisin A and its bioengineered derivatives [61]. The value of these compounds has moved beyond simply removing biofilms from surfaces, and instead, they represent a promising potential in the expansion of bio-preservatives as an alternative to harsh chemically processed food additives [61]. Nisin is one of the most well-known of the bacteriocins studied and has already been approved as a natural food preservative, but the emergence of nisin-resistant strains has already begun to occur [62,63]. Nisin A is already used in Nisaplin commercial food preservative products, but the addition of certain essential oil combinations, such as cinnamaldehyde and citric acid, to bioengineered purified peptide derivatives of nisin A has proven to be an even more cost-effective option requiring far less of the active components currently used in Nisaplin [61]. This enhancement of the already promoted natural products successfully inhibits and eradicates *Listeria* biofilm growth and helps overcome the already emerging nisin-resistant mutants while also allowing for lower concentrations of additives in food matrices [61]. Nisin has also shown synergistic enhancement with essential oil compounds linalool and p-coumaric acid against planktonic cells and preformed biofilms of *B. cereus* and *S. typhimurium* [64]. These results are very promising for the food industry, as they demonstrate effectiveness against both Gram-positive and Gram-negative foodborne bacteria [64]. Ultimately, it should be noted that not all essential oils are created equally, so to speak. It is important to thoroughly test essential oils against various strains and in combination with each other to determine the most effective compounds. For instance, in one study, a synergistic combination of cinnamaldehyde and eugenol was highly effective against preformed foodborne biofilms of *L. monocytogenes* and *S. typhimurium*, whereas β-caryophyllene was unsuccessful in degrading the same biofilms [65]. Another consideration to make when considering the use of essential oils in food products is the potential for allergic reactions [66]. In one study exploring the use of citrus essential oils for food product use, some instances of skin irritation and allergic reactions were observed [66]. As most studies performed on essential oil used in food processing have been in vitro and lack clinical testing results, it is important to note the current limitations in usage potential, as well as the necessary regulatory and FDA guidelines that would be necessary to incorporate these compounds into marketed food items [67].

## 4. Gaseous and Aqueous Control, as Well as Photocatalysis

Developments in the food industry have allowed for the mass production of crops year-round and plant-based alternatives to meat but has yet to fully solve the issue of microbial contamination [68]. Various pathogenic bacteria like *Listeria monocytogenes* infiltrate food and form biofilms that are difficult to remove due to their high endurance [68]. Due to this, attention has been placed on finding a variety of ways to treat foodborne biofilms, especially treatment options involving natural compounds [68].

**Figure 2:**
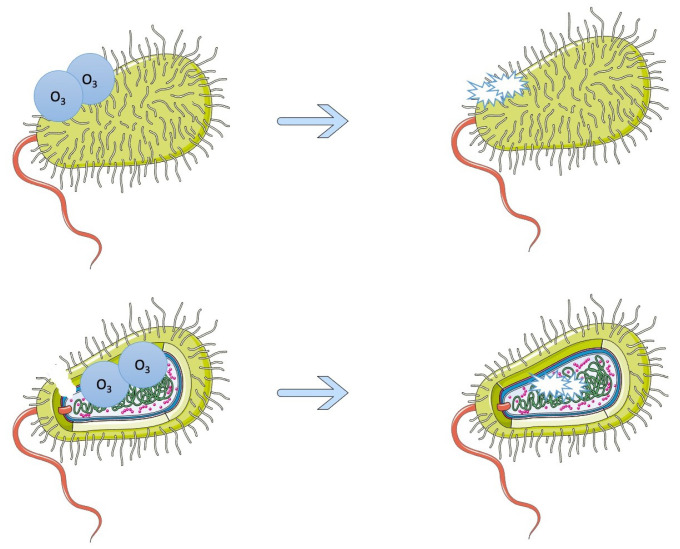
The effects of ozone on bacterial cells. Ozone’s oxidative potential breaks down the bacterium’s cell wall and is then able to degrade the proteins inside the cell, preventing it from functioning and replicating.

One way of treating foodborne biofilms naturally is with gaseous ozone, which does not result in harmful residues on food and because of its oxidative potential can degrade peptides and fatty acids, damaging bacterial cells [68] (Figure 2). Various studies have shown the effectiveness of gaseous ozone on biofilms, with one examining the effects on *Pseudomonas fluorescens*, *Staphylococcus aureus*, and *L. monocytogenes* specifically [69]. Different concentrations of gaseous ozone were used on the biofilms, and, at the highest concentrations (0.2 ppm to 20 ppm), there was a total inactivation of *L. monocytogenes* and reductions of the *P. fluorescens* and *S. aureus* biofilms [69]. Another study on the effect of gaseous ozone on food-related *L. monocytogenes* strains showed microbial load reductions, and in 59% of the strains, there was a significant decrease in biofilm biomass [70]. An additional study also saw that gaseous ozone decreased biofilm production of *L. monocytogenes* [71]. The inhibiting effects of gaseous ozone were seen in *Pseudomonas* spp. strains as well and acted on weak and moderate/strong strains [68]. However, on all strains except for one, the gaseous ozone could not effectively eradicate the biofilms [68]. This, and the previous studies mentioned, show that gaseous ozone can be helpful in inhibiting foodborne biofilm growth but cannot alone completely eradicate them, so it would likely have to be used in tandem with another treatment option [68,70,71] One example of this type of treatment is the combination of citric acid and gaseous ozone, which was tested on *Acinetobacter baumannii* and decreased the number of viable bacteria with a 99.99% inhibition rate [72]. Another type of gaseous treatment that has been researched is using gaseous chlorine dioxide in a 10-min treatment, which, in one study, caused a 3.21 log_10_ CFU/cm^2^ reduction in L. monocytogenes [73]. In addition, there is the method of aerosolized sodium hypochlorite and peracetic acid, which was tested on biofilms made up of strains of *E. coli*, *S. Typhimurium*, and *L. monocytogenes* [74]. After 50 min of treatment with 100 ppm sodium hypochlorite and peracetic acid, the biofilm cells were significantly reduced [74]. After 10 to 30 min of 200 and 400 ppm peracetic acid, there was a reduction of biofilm cells to less than the detection limit [74].

Research on aqueous methods of treating foodborne biofilms has also been produced with a focus on aqueous ozone [69]. In one study on biofilms of *P. fluorescens*, *S. aureus*, and *L. monocytogenes*, there was a reduction of 3.26 to 5.23 Log CFU/cm^2^ after 20 min of aqueous ozone treatment in dynamic conditions [69]. In comparison to gaseous ozone, aqueous ozone tends to be more effective and requires shorter treatment time [75]. Other studies examined the effects of aqueous sodium hypochlorite and observed that cells during starvation responded to it [76]. Aqueous chlorine dioxide was also used in an attempt to inhibit the biofilms of *E. coli* and resulted in a reduction in the number of *E. coli* [77]. The number continued to decrease as the surface with the biofilm dried, showing that aqueous chlorine dioxide continues to kill bacteria even after the original treatment [77]. In this experiment, aqueous sodium hypochlorite was also used to treat the biofilms, but it was not as effective, and its ability to reduce the amount of bacteria depended on the type of surface the biofilm was placed on [77]. Another study observed the effect of acidic electrolyzed water made from different sodium chloride concentrations and found that these solutions eradicated *Vibrio parahaemolyticus* and *L. monocytogenes* biofilms [78]. All levels of sodium chloride concentration had an effect on the biofilms, but the higher the concentration the more the cell number decreased [78].

An additional method of controlling foodborne biofilms is by using ultrasound [79]. Ultrasounds are able to eradicate biofilms because of the oscillation they produce, and, in a study, a flat ultrasonic transducer was able to remove *E. coli* and *S. aureus* milk biofilms [79]. Low-frequency ultrasound is also able to reduce the biofilm biomass of L. monocytogenes by 87% and decrease the number of viable cells in the biofilm [79]. Ultrasound has also been used in combination with peracetic acid to reduce the number of cells in an *S. enterica* biofilm [79]. Another similar combination is X-ray irradiation and aqueous chlorine dioxide, which reduced *S. enterica* biofilms on quail eggshells [80].

Another strategy for inhibiting or removing foodborne biofilms is photocatalysis [81,82,83]. There are a variety of photocatalytic treatments, with a prominent one being the photoactivation of a titanium dioxide coating [81]. Titanium dioxide is especially useful for foodborne biofilms, as it is already used in many human foods and food contact materials [82]. During this activation, reactive oxygen species, such as hydroxyl radicals, form, which can cause the breakdown of membranes, inhibit growth, and kill the cells [81,84]. In one study, *E. coli* and *S. typhimurium* on steel surfaces were inhibited by the coatings and UV radiation after five minutes, and *L. monocytogenes* was inhibited after 10 min [81]. For all coated surfaces, the UV radiation led to significant viability reduction when compared to surfaces that were not coated [81]. The efficiency of the treatment was affected by the type of bacteria, as Gram-positive bacteria were more resistant due to their different membrane architecture [81]. The type of surface also influences efficiency, with steel surfaces resulting in greater inhibition than aluminum surfaces [81,82]. In other studies, it was shown that glass surfaces had higher efficiency than steel, as it took 120 min of UVA irradiation for titanium-dioxide-coated glass surfaces to no longer have a detectable biofilm population, while for steel surfaces it took 180 min [82]. An additional variable to consider when photoactivating nanoparticles is whether the photocatalysts are suspended or immobilized [85]. One study observed that after 180 min of irradiation with suspended titanium dioxide, there was a 6-log10 (CFU/cm^2^) reduction in *S. Typhimurium* cell density, but with the same amount of time, immobilized titanium dioxide resulted in a 4-log10 (CFU/cm^2^) reduction [85]. This is likely because immobilized photocatalysts usually cover a small portion of the bacterial cell wall, while those in suspension can cover more of the cell wall [85]. A potential limitation for photocatalytic treatment with titanium dioxide is that, in a study, it did not cause a change in the quantity of the surviving biofilm after the treatment [84].

Research for photocatalysis as a way to limit biofilm formation has also been carried out using graphitic carbon nitride–chitosan composites as photocatalysts [86]. Similar to titanium dioxide, it also works by resulting in reactive oxygen species [86]. The effects of this were displayed in a study wherein the composites were under continuous white LED light irradiation and it resulted in the complete inhibition of the biofilms of *Staphylococcus epidermidis*, *P. aeruginosa* PAO1, and *E. coli* O157: H7 [86]. This method was also able to significantly remove the biomass of mature biofilms of *S. epidermidis* and *P. aeruginosa* PAO1 but not for *E. coli* O157: H7 [86]. Other strategies involved using 5-nitroindole-capped bimetal (copper and zinc) nanoparticles as photocatalysts [87]. These photocatalysts led to a 6.1 log reduction of the *Enterobacter tabaci* strain MBR1 and a 6 log reduction in *S. aureus* ATCC6538 cell density after 120 min in one study [87]. There was also an observed increase in bactericidal activity when comparing the effects of the 5-nitrodole-capped nanoparticles alone versus with the light irradiation [87].

## 5. Enzymatic Treatment

Enzymatic treatment is another potential means to target foodborne biofilms [88,89,90,91,92,93,94]. A variety of enzymes have been tested on different biofilms in an attempt to inhibit or remove it [88,89,90,91,92,93,94]. There is not a complete understanding yet of how enzymes can inhibit biofilms. One hypothesized mechanism is that enzymes might be able to break up the matrix of extracellular polymeric substances (EPS) [89]. One enzyme that was specifically studied was Flavourzyme, which is a combination of different endo and exopeptidases with amylase [88]. Flavourzyme is active in a large range of pH and temperatures but is costly [88]. An experiment with Flavourzyme showed that sub-minimum inhibitory concentrations of it resulted in a 4.0 and 5.5 log inhibition of *S. Typhimurium* and *E. coli* biofilm formation after a twenty-four-hour treatment [88]. The specific concentrations to prevent growth were 350 and 300 μL/mL, and at 500 μL/mL, it resulted in a bactericidal effect [88]. Flavourzyme is already used in food often, which reduces safety concerns over using it as an antibiofilm substance [88]. Enzymes tested in other studies were α-amylase, amyloglucosidase, cellulase, DNase, proteinase K, and a combination of proteinase K and chlorine [89]. Proteinase K was more successful compared to the others at inhibiting growth and breaking down the mature biofilm matrix [89]. There was also an observed difference from treating with proteinase K alone versus with chlorine, as cells of a *S. Typhimurium* biofilm were synergistically inactivated by the combination in comparison to the proteinase K alone [89]. This shows the potential of enzymatic treatments helping sanitizers inhibit or remove biofilm cells, as the matrices of EPSs are heterogenic, so they may require multiple enzymes or sanitizers [89]. The effects of proteinase K were also examined in another study along with DNase I, cellulase, and NaCIO against an *E. coli* O157:H7 biofilm [90]. All of the enzymes resulted in a significant reduction (16.–36.7%) in the biofilm matrix, but the sequential treatment of proteinase K followed by NaCIO led to a much higher reduction [90]. This again illustrates how enzymes can make biofilms less resistant to sanitizers [90]. Another enzymatic treatment that was used experimentally was a mixture of ethoxylated sodium lauryl ether glycolate, *N*-oxide *N*,N-dimethyl-C12-C14-alkylamine, anionic surfactants, nonionic surfactants, enzymes including proteases, and phenoxyethanol [91]. It led to a maximum reduction of 6.9 log CFU/cm2 for *L. monocytogenes* 5672 and for multiple strains significantly reduced the cellular load [91]. It also caused a 85−99% detachment of the mature biofilm for the different strains [91]. The variety between strains illustrates that the effectiveness depends on the strain, as they have slightly different structures [91]. A different study examined how a preventive enzymatic treatment (protease (5.0%), lipase (0.5%), amylase (2.5%)) and an aggressive enzymatic treatment (protease (10.0%), lipase (1.0%), amylase (5.0%)) affected *S. enterica* serovar *Typhimurium* and *Cronobacter sakazakii* biofilms [92]. Both treatments resulted in significant reductions of microbial load with a maximum log reduction of 3 log CFU cm^−2^, but the aggressive treatment was more successful than the preventive treatment [92].

The potential for enzymatic treatment was also observed in a study using nucleolytic enzymes from bacterium *Cobetia amphilecti*: CmNuc (similar to nuclease), CmEEP (similar to DNAase), and CmAP (alkaline phosphatase) [93]. CmEEP and CmNUC were species-independent and broke down biofilms at low pH [93]. CmAP’s effect was dose-dependent, and it resulted in the complete removal of the *P. aeruginosa* extracellular matrix in the area it was placed after twelve hours [93]. CmAP also had an enzyme concentration of 1.1 μg/mL of the protein, with the specific activity of 2300 μ/mg as the maximum effective antimicrobial dose [93]. This would allow food safety to become less costly [93]. Surfactants, bio-enzymes, and a combination of both were also tested in one study on a multistrain cocktail of *Salmonella* [94]. The surfactants were cetyltrimethyl ammonium bromide (CTAB), sodium dodecyl sulfate (SDS), rhamnolipid, and tween-80, while the bio-enzymes were proteinase K, dispase II, subtilisin, cellulase, and glucoside amylase [94]. CTAB and SDS resulted in a greater reduction of biofilm cells compared to the other surfactants, while cellulase resulted in a greater reduction compared to the other enzymes [94]. For both the surfactants and enzymes, the reduction was affected by the concentration of either [94]. However, the surfactants overall led to a higher biofilm reduction than the enzymes [94]. The treatment strategy of CTAB followed by cellulase was very effective and removed 100% of mature biofilm, showing that a combination of treatments is more likely to successfully treat biofilms [94].

## 6. Conclusions

The food industry is a major aspect of everyday life and global economies, and yet it also remains highly vulnerable to dangerous contamination from foodborne pathogens that accumulate as biofilms and result in spoilage as well as financial losses [95]. Although estimates vary, some sources cite approximately 600 million illnesses and 420,000 deaths globally each year from contaminated food, marking approximately 7.5% of annual deaths [96]. Financial devastation also becomes evident in various estimates, including one study using the USDA Economic Research Service, which estimated the economic cost of 15 major foodborne illnesses to be USD 15.5 billion in 2013 and USD 17.6 billion in 2018 [97]. In the food industry, biofilms present a unique challenge for removal and prevention, as they provide distinctive protection layers for microbes and tend to include a diverse range of antibiotic-resistant organisms [98]. Natural means of removal and prevention would not only allow for a more effective means of minimizing the emergence of resistant strains but would also provide ways to increase the nutritional or health values of food products at the same time [99,100]. Furthermore, natural options, such as essential oils, provide opportunities for more powerful synergistic effects without the dangers of noxious odors or harmful toxins that are standard with more traditional highly potent chemicals [60]. Whereas most studies focus on the benefits of natural means of eradicating biofilms on food processing surfaces, it is also important to note that natural compounds have also shown great promise as food-grade additives to destroy biofilms that integrate into the food matrix [61]. Overall, it is important to consider the advantages and disadvantages of the emerging natural options (Table 1) and determine the optimal means to maximize foodborne biofilm eradication while avoiding costly or harmful side effects.

## Figures and Tables

**Table 1:** Summary of advantages and disadvantages of natural methods to control foodborne biofilms.

Method	Advantages	Disadvantages	References
**Bacteria** **(Bacteriocins)**	■ Potent antimicrobial activity ■ Strong tolerance to acid and bile gastric conditions ■ Adds properties to enhance food production, such as fermentation ■ Reduces dangerous chemicals and is categorized as environmentally sound ■ Large reservoir due to continual discovery of novel bacteria ■ Enhancements, such as nanoparticle vesicles or synergy, increase bioavailability and efficacies ■ Cytotoxicity assays demonstrated not toxic to animal cells	■ Some categories, such as class IV, contain glycoproteins that are susceptible to amylase and proteolytic enzymes	[6,7,8,9,10,11,12]
**Bacteriophages**	■ High specificity and versatility ■ High stability ■ Kosher, organic, and halal certified ■ Promising pH and temperature range of efficacy ■ Able to select for strains lacking any antibiotic-resistance genes, virulence factor genes, or integrases ■ Broad spectrum lytic activity ■ Reduces reliance on antibiotics	■ Highly variable efficacy depends on many factors, such as strains, temperature, MOI, biofilm content, and surfaces ■ Emergence of phage resistance possible ■ Pathogenicity gene transmission a concern ■ Progeny viruses lack efficacy of initial viruses. ■ Release of endotoxins from Gram-negative bacteria ■ Unknown ecological perturbation effects ■ Consumer acceptance issues	[13,14,15,16,17,18,19,20,21,22,23,24,25,26,27]
**Fungi**	■ Useful extracellular thermostable peptides extracted ■ Peptide functionality at wide pH and temperature ranges ■ Nontoxic to human red blood cells ■ Fungi are able to mass produce biosynthesized nanoparticles	■ Less research has been done on the use of fungi, so disadvantages are not well-established yet.	[31,32,33,34,35,36,37]
**Phytochemicals, Plant Extracts, and Essential Oils**	■ Effective to reduce or prevent quorum-sensing and motility activities in bacteria ■ Already demonstrated safe in treatment of human ailments ■ Additional valuable properties, such as anti-cancer and anti-inflammatory abilities ■ Potent antioxidant properties ■ Effective against multidrug-resistant pathogens ■ Synergistic effects	■ Exact mechanisms remain elusive ■ Requires further molecular testing ■ Variability in effectiveness ■ Must be screened for potential cytotoxic effects	[38,39,40,41,42,43,44,45,46,47,48,49,50,51,52,53,54,55,56,57,58,59,60,61,62,63,64,65,66,67]
**Gaseous and Aqueous**	■ Oxidative activities ■ Does not leave harmful residues ■ Continues to kill after initial treatment	■ Inhibits but does not completely eradicate biofilm, so likely requires in tandem use with other options	[69,70,71,72,73,74,75,76,77,78]
**Enzymatic**	■ Active at large range of conditions ■ Already used in foods so fewer safety concerns	■ Some are costly ■ Varying efficacy depending on the enzyme	[88,89,90,91,92,93,94]

## Data Availability

Not applicable.

## References

[1] Aryal M., Muriana P.M. (2019). Efficacy of Commercial Sanitizers Used in Food Processing Facilities for Inactivation of Listeria Monocytogenes, *E. Coli* O157:H7, and Salmonella Biofilms. Foods.

[2] Iñiguez-Moreno M., Gutiérrez-Lomelí M., Avila-Novoa M.G. (2019). Kinetics of Biofilm Formation by Pathogenic and Spoilage Microorganisms under Conditions That Mimic the Poultry, Meat, and Egg Processing Industries. Int. J. Food Microbiol..

[3] Rather M., Gupta K., Bardhan P., Borah M., Sarkar A., Eldiehy K., Bhuyan S., Mandal M. (2021). Microbial Biofilm: A Matter of Grave Concern for Human Health and Food Industry. J. Basic Microbiol..

[4] Bridier A., Sanchez-Vizuete P., Guilbaud M., Piard J.-C., Naïtali M., Briandet R. (2015). Biofilm-Associated Persistence of Food-Borne Pathogens. Food Microbiol..

[5] Oxaran V., Dittmann K.K., Lee S.H.I., Chaul L.T., Fernandes de Oliveira C.A., Corassin C.H., Alves V.F., De Martinis E.C.P., Gram L. (2018). Behavior of Foodborne Pathogens Listeria Monocytogenes and Staphylococcus Aureus in Mixed-Species Biofilms Exposed to Biocides. Appl. Environ. Microbiol..

[6] Gavrilova E., Anisimova E., Gabdelkhadieva A., Nikitina E., Vafina A., Yarullina D., Bogachev M., Kayumov A. (2019). Newly Isolated Lactic Acid Bacteria from Silage Targeting Biofilms of Foodborne Pathogens during Milk Fermentation. BMC Microbiol..

[7] Kim N.-N., Kim W.J., Kang S.-S. (2019). Anti-Biofilm Effect of Crude Bacteriocin Derived from Lactobacillus Brevis DF01 on Escherichia Coli and Salmonella Typhimurium. Food Control.

[8] Kang T.-K., Kim W.-J. (2010). Characterization of an Amylase-Sensitive Bacteriocin DF01 Produced by Lactobacillus Brevis DF01 Isolated from Dongchimi, Korean Fermented Vegetable. Korean J. Food Sci. Anim. Resour..

[9] Tatsaporn T., Kornkanok K. (2020). Using Potential Lactic Acid Bacteria Biofilms and Their Compounds to Control Biofilms of Foodborne Pathogens. Biotechnol. Rep..

[10] Bindiya E.S., Tina K.J., Sasidharan R.S., Bhat S.G. (2019). BaCf3: Highly Thermostable Bacteriocin from Bacillus Amyloliquefaciens BTSS3 Antagonistic on Food-Borne Pathogens. 3 Biotech.

[11] Niaz T., Shabbir S., Noor T., Imran M. (2019). Antimicrobial and Antibiofilm Potential of Bacteriocin Loaded Nano-Vesicles Functionalized with Rhamnolipids against Foodborne Pathogens. LWT.

[12] Turgis M., Vu K.D., Dupont C., Lacroix M. (2012). Combined Antimicrobial Effect of Essential Oils and Bacteriocins against Foodborne Pathogens and Food Spoilage Bacteria. Food Res. Int..

[13] Sadekuzzaman M., Yang S., Mizan M.F.R., Ha S.-D. (2017). Reduction of Escherichia coli O157:H7 in Biofilms Using Bacteriophage BPECO 19. J. Food Sci..

[14] Garvey M. (2022). Bacteriophages and Food Production: Biocontrol and Bio-Preservation Options for Food Safety. Antibiotics.

[15] Meaden S., Koskella B. (2013). Exploring the Risks of Phage Application in the Environment. Front Microbiol..

[16] Moye Z.D., Woolston J., Sulakvelidze A. (2018). Bacteriophage Applications for Food Production and Processing. Viruses.

[17] Guenther S., Herzig O., Fieseler L., Klumpp J., Loessner M.J. (2012). Biocontrol of Salmonella Typhimurium in RTE Foods with the Virulent Bacteriophage FO1-E2. Int. J. Food Microbiol..

[18] Kang H.-W., Kim J.-W., Jung T.-S., Woo G.-J. (2013). Wksl3, a New Biocontrol Agent for Salmonella Enterica Serovars Enteritidis and Typhimurium in Foods: Characterization, Application, Sequence Analysis, and Oral Acute Toxicity Study. Appl. Env. Microbiol..

[19] Endersen L., Coffey A. (2020). The Use of Bacteriophages for Food Safety. Curr. Opin. Food Sci..

[20] Islam M.S., Zhou Y., Liang L., Nime I., Liu K., Yan T., Wang X., Li J. (2019). Application of a Phage Cocktail for Control of Salmonella in Foods and Reducing Biofilms. Viruses.

[21] Rohwer F., Segall A.M. (2015). A Century of Phage Lessons. Nature.

[22] Zhu W., Ding Y., Huang C., Wang J., Wang J., Wang X. (2022). Genomic Characterization of a Novel Bacteriophage STP55 Revealed Its Prominent Capacity in Disrupting the Dual-Species Biofilm Formed by Salmonella Typhimurium and Escherichia Coli O157: H7 Strains. Arch. Microbiol..

[23] Yew C.-H.T., Gurumoorthy N., Nordin F., Tye G.J., Zaman W.S.W.K., Tan J.J., Ng M.H. (2022). Integrase Deficient Lentiviral Vector: Prospects for Safe Clinical Applications. PeerJ.

[24] Vikram A., Woolston J., Sulakvelidze A. (2021). Phage Biocontrol Applications in Food Production and Processing. Curr. Issues Mol. Biol..

[25] Gdoura-Ben Amor M., Culot A., Techer C., AlReshidi M., Adnan M., Jan S., Baron F., Grosset N., Snoussi M., Gdoura R. (2022). Isolation, Partial Characterization and Application of Bacteriophages in Eradicating Biofilm Formation by Bacillus Cereus on Stainless Steel Surfaces in Food Processing Facilities. Pathogens.

[26] Byun K.H., Han S., Choi M., Kim B.-H., Park S.H., Ha S.-D. (2022). Biofilm Eradication Ability of Phage Cocktail against Listeria Monocytogenes Biofilms Formed on Food Contact Materials and Effect on Virulence-Related Genes and Biofilm Structure. Food Res. Int..

[27] Cepko L.C.S., Garling E.E., Dinsdale M.J., Scott W.P., Bandy L., Nice T., Faber-Hammond J., Mellies J.L. (2020). Myoviridae Phage PDX Kills Enteroaggregative Escherichia Coli without Human Microbiome Dysbiosis. J. Med. Microbiol..

[28] Kapila Y.L. (2021). Oral Health’s Inextricable Connection to Systemic Health: Special Populations Bring to Bear Multimodal Relationships and Factors Connecting Periodontal Disease to Systemic Diseases and Conditions. Periodontology.

[29] Esposito A.M., Esposito M.M., Ptashnik A. (2022). Phylogenetic Diversity of Animal Oral and Gastrointestinal Viromes Useful in Surveillance of Zoonoses. Microorganisms.

[30] Cao J., Wang C., Zhang Y., Lei G., Xu K., Zhao N., Lu J., Meng F., Yu L., Yan J. (2021). Integrated Gut Virome and Bacteriome Dynamics in COVID-19 Patients. Gut Microbes.

[31] Vunduk J., Wan-Mohtar W.A.A.Q.I., Mohamad S.A., Abd Halim N.H., Mohd Dzomir A.Z., Žižak Ž., Klaus A. (2019). Polysaccharides of Pleurotus Flabellatus Strain Mynuk Produced by Submerged Fermentation as a Promising Novel Tool against Adhesion and Biofilm Formation of Foodborne Pathogens. LWT.

[32] Klaus A., Kozarski M., Vunduk J., Todorovic N., Jakovljevic D., Zizak Z., Pavlovic V., Levic S., Niksic M., Van Griensven L.J.L.D. (2015). Biological Potential of Extracts of the Wild Edible Basidiomycete Mushroom Grifola Frondosa. Food Res. Int..

[33] Fasciana T., Gargano M.L., Serra N., Galia E., Arrigo I., Tricoli M.R., Diquattro O., Graceffa G., Vieni S., Venturella G. (2021). Potential Activity of Albino Grifola Frondosa Mushroom Extract against Biofilm of Meticillin-Resistant Staphylococcus Aureus. J. Fungi.

[34] Aqueous Extracts of Wild Mushrooms Show Antimicrobial and Antiadhesion Activities against Bacteria and Fungi—Klančnik—2017—Phytotherapy Research—Wiley Online Library. https://onlinelibrary.wiley.com/doi/abs/10.1002/ptr.5934.

[35] Schillaci D., Arizza V., Gargano M.L., Venturella G. (2013). Antibacterial Activity of Mediterranean Oyster Mushrooms, Species of Genus *Pleurotus* (Higher Basidiomycetes). IJM.

[36] Kollakalnaduvil Raghavan R.M., Ali Pannippara M., Kesav S., Mathew A., Bhat G.S., Hatha AA M., Kk E. (2021). MFAP9: Characterization of an Extracellular Thermostable Antibacterial Peptide from Marine Fungus with Biofilm Eradication Potential. J. Pharm. Biomed. Anal..

[37] Green Synthesized Silver Nanoparticles by Marine Endophytic Fungus Penicillium Polonicum and Its Antibacterial Efficacy against Biofilm Forming, Multidrug-Resistant Acinetobacter Baumanii—ScienceDirect. https://www.sciencedirect.com/science/article/abs/pii/S0882401017315127.

[38] Rahman M.R.T., Lou Z., Yu F., Wang P., Wang H. (2017). Anti-Quorum Sensing and Anti-Biofilm Activity of Amomum Tsaoko (Amommum Tsao-Ko Crevost et Lemarie) on Foodborne Pathogens. Saudi J. Biol. Sci..

[39] Hammer B.K., Bassler B.L. (2003). Quorum Sensing Controls Biofilm Formation in Vibrio Cholerae. Mol. Microbiol..

[40] Inés Molina R.D., Campos-Silva R., Díaz M.A., Macedo A.J., Blázquez M.A., Alberto M.R., Arena M.E. (2020). Laurel Extracts Inhibit Quorum Sensing, Virulence Factors and Biofilm of Foodborne Pathogens. LWT.

[41] Ramos C., Teixeira B., Batista I., Matos O., Serrano C., Neng N.R., Nogueira J.M.F., Nunes M.L., Marques A. (2012). Antioxidant and Antibacterial Activity of Essential Oil and Extracts of Bay Laurel Laurus Nobilis Linnaeus (Lauraceae) from Portugal. Nat. Prod. Res..

[42] Yilmaz E.S., Timur M., Aslim B. (2013). Antimicrobial, Antioxidant Activity of the Essential Oil of Bay Laurel from Hatay, Turkey. J. Essent. Oil Bear. Plants.

[43] Ordoudi S.A., Papapostolou M., Nenadis N., Mantzouridou F.T., Tsimidou M.Z. (2022). Bay Laurel (*Laurus Nobilis* L.) Essential Oil as a Food Preservative Source: Chemistry, Quality Control, Activity Assessment, and Applications to Olive Industry Products. Foods.

[44] Somrani M., Inglés M.-C., Debbabi H., Abidi F., Palop A. (2020). Garlic, Onion, and Cinnamon Essential Oil Anti-Biofilms’ Effect against Listeria Monocytogenes. Foods.

[45] Casella S., Leonardi M., Melai B., Fratini F., Pistelli L. (2013). The Role of Diallyl Sulfides and Dipropyl Sulfides in the In Vitro Antimicrobial Activity of the Essential Oil of Garlic, *Allium Sativum* L., and Leek, *Allium Porrum* L.. Phytother. Res..

[46] Ross Z.M., O’Gara E.A., Hill D.J., Sleightholme H.V., Maslin D.J. (2001). Antimicrobial Properties of Garlic Oil against Human Enteric Bacteria: Evaluation of Methodologies and Comparisons with Garlic Oil Sulfides and Garlic Powder. Appl. Env. Microbiol..

[47] Ye H., Shen S., Xu J., Lin S., Yuan Y., Jones G.S. (2013). Synergistic Interactions of Cinnamaldehyde in Combination with Carvacrol against Food-Borne Bacteria. Food Control.

[48] Zhou F., Ji B., Zhang H., Jiang H., Yang Z., Li J., Li J., Yan W. (2007). The Antibacterial Effect of Cinnamaldehyde, Thymol, Carvacrol and Their Combinations Against the Foodborne Pathogen Salmonella Typhimurium. J. Food Saf..

[49] Siddiqua S., Anusha B.A., Ashwini L.S., Negi P.S. (2015). Antibacterial Activity of Cinnamaldehyde and Clove Oil: Effect on Selected Foodborne Pathogens in Model Food Systems and Watermelon Juice. J. Food Sci. Technol..

[50] Nowotarska S.W., Nowotarski K., Grant I.R., Elliott C.T., Friedman M., Situ C. (2017). Mechanisms of Antimicrobial Action of Cinnamon and Oregano Oils, Cinnamaldehyde, Carvacrol, 2,5-Dihydroxybenzaldehyde, and 2-Hydroxy-5-Methoxybenzaldehyde against Mycobacterium Avium Subsp. Paratuberculosis (Map). Foods.

[51] Cinici E., Dilekmen N., Kutlu Z., Dincer B., Cinici O., Balta H., Calik I. (2020). Carvacrol Protects against Paclitaxel-Induced Retinal and Optic Nerve Cytotoxicity: A Histopathological Study. Beyoglu Eye J..

[52] Nostro A., Roccaro A.S., Bisignano G., Marino A., Cannatelli M.A., Pizzimenti F.C., Cioni P.L., Procopio F., Blanco A.R. (2007). Effects of Oregano, Carvacrol and Thymol on Staphylococcus Aureus and Staphylococcus Epidermidis Biofilms. J. Med. Microbiol..

[53] Aristatile B., Al-Numair K.S., Veeramani C., Pugalendi K.V. (2009). Effect of Carvacrol on Hepatic Marker Enzymes and Antioxidant Status in D-Galactosamine-Induced Hepatotoxicity in Rats. Fundam. Clin. Pharmacol..

[54] Baranauskaite J., Kubiliene A., Marksa M., Petrikaite V., Vitkevičius K., Baranauskas A., Bernatoniene J. (2017). The Influence of Different Oregano Species on the Antioxidant Activity Determined Using HPLC Postcolumn DPPH Method and Anticancer Activity of Carvacrol and Rosmarinic Acid. BioMed. Res. Int..

[55] Bukovská A., Cikoš Š., Juhás Š., Il’ková G., Rehák P., Koppel J. (2007). Effects of a Combination of Thyme and Oregano Essential Oils on TNBS-Induced Colitis in Mice. Mediat. Inflamm..

[56] Sadekuzzaman M., Mizan M.F.R., Kim H.-S., Yang S., Ha S.-D. (2018). Activity of Thyme and Tea Tree Essential Oils against Selected Foodborne Pathogens in Biofilms on Abiotic Surfaces. LWT.

[57] De-Montijo-Prieto S., Razola-Díaz M.d.C., Gómez-Caravaca A.M., Guerra-Hernandez E.J., Jiménez-Valera M., Garcia-Villanova B., Ruiz-Bravo A., Verardo V. (2021). Essential Oils from Fruit and Vegetables, Aromatic Herbs, and Spices: Composition, Antioxidant, and Antimicrobial Activities. Biology.

[58] Quendera A.P., Barreto A.S., Semedo-Lemsaddek T. (2019). Antimicrobial Activity of Essential Oils against Foodborne Multidrug-Resistant Enterococci and Aeromonads in Planktonic and Biofilm State. Food Sci. Technol. Int..

[59] Moussaoui F., Alaoui T. (2016). Evaluation of Antibacterial Activity and Synergistic Effect between Antibiotic and the Essential Oils of Some Medicinal Plants. Asian Pac. J. Trop. Biomed..

[60] Liu Q., Niu H., Zhang W., Mu H., Sun C., Duan J. (2015). Synergy among Thymol, Eugenol, Berberine, Cinnamaldehyde and Streptomycin against Planktonic and Biofilm-Associated Food-Borne Pathogens. Lett. Appl. Microbiol..

[61] Smith M.K., Draper L.A., Hazelhoff P.-J., Cotter P.D., Ross R.P., Hill C. (2016). A Bioengineered Nisin Derivative, M21A, in Combination with Food Grade Additives Eradicates Biofilms of Listeria Monocytogenes. Front. Microbiol..

[62] Barbosa A.A.T., de Melo M.R., da Silva C.M.R., Jain S., Dolabella S.S. (2021). Nisin Resistance in Gram-Positive Bacteria and Approaches to Circumvent Resistance for Successful Therapeutic Use. Crit. Rev. Microbiol..

[63] Stincone P., Miyamoto K.N., Timbe P.P.R., Lieske I., Brandelli A. (2020). Nisin Influence on the Expression of Listeria Monocytogenes Surface Proteins. J. Proteom..

[64] Bag A., Chattopadhyay R.R. (2017). Synergistic Antibacterial and Antibiofilm Efficacy of Nisin in Combination with P-Coumaric Acid against Food-Borne Bacteria Bacillus Cereus and Salmonella Typhimurium. Lett. Appl. Microbiol..

[65] Purkait S. (2020). Evaluation of Antibiofilm Efficacy of Essential Oil Components Β-caryophyllene, Cinnamaldehyde and Eugenol Alone and in Combination against Biofilm Formation and Preformed Biofilms of Listeria Monocytogenes and Salmonella Typhimurium. Lett. Appl. Microbiol..

[66] Bora H., Kamle M., Mahato D.K., Tiwari P., Kumar P. (2020). Citrus Essential Oils (CEOs) and Their Applications in Food: An Overview. Plants.

[67] Valdivieso-Ugarte M., Gomez-Llorente C., Plaza-Díaz J., Gil Á. (2019). Antimicrobial, Antioxidant, and Immunomodulatory Properties of Essential Oils: A Systematic Review. Nutrients.

[68] Panebianco F., Rubiola S., Chiesa F., Civera T., Di Ciccio P.A. (2022). Effect of Gaseous Ozone Treatment on Biofilm of Dairy-Isolated Pseudomonas Spp. Strains. Ital. J. Food Saf..

[69] Marino M., Maifreni M., Baggio A., Innocente N. (2018). Inactivation of Foodborne Bacteria Biofilms by Aqueous and Gaseous Ozone. Front. Microbiol..

[70] Panebianco F., Rubiola S., Chiesa F., Civera T., Di Ciccio P.A. (2021). Effect of Gaseous Ozone on Listeria Monocytogenes Planktonic Cells and Biofilm: An In Vitro Study. Foods.

[71] Di Ciccio P., Ghidini S., Zanardi E., Borrello S., Vergara A., Festino A., Ianieri A. (2014). Effects of Gaseous Ozone on Food-Borne Pathogens. Ital. J. Food Sci..

[72] Piletić K., Kovač B., Planinić M., Vasiljev V., Karačonji I.B., Žigon J., Gobin I., Oder M. (2022). Combined Biocidal Effect of Gaseous Ozone and Citric Acid on Acinetobacter Baumannii Biofilm Formed on Ceramic Tiles and Polystyrene as a Novel Approach for Infection Prevention and Control. Processes.

[73] Vaid R., Linton R.H., Morgan M.T. (2010). Comparison of Inactivation of Listeria Monocytogenes within a Biofilm Matrix Using Chlorine Dioxide Gas, Aqueous Chlorine Dioxide and Sodium Hypochlorite Treatments. Food Microbiol..

[74] Park S.-H., Cheon H.-L., Park K.-H., Chung M.-S., Choi S.H., Ryu S., Kang D.-H. (2012). Inactivation of Biofilm Cells of Foodborne Pathogen by Aerosolized Sanitizers. Int. J. Food Microbiol..

[75] Panebianco F., Rubiola S., Di Ciccio P.A. (2022). The Use of Ozone as an Eco-Friendly Strategy against Microbial Biofilm in Dairy Manufacturing Plants: A Review. Microorganisms.

[76] Zhao X., Zhao F., Wang J., Zhong N. (2017). Biofilm Formation and Control Strategies of Foodborne Pathogens: Food Safety Perspectives. RSC Adv..

[77] Bang J., Hong A., Kim H., Beuchat L.R., Rhee M.S., Kim Y., Ryu J.-H. (2014). Inactivation of Escherichia Coli O157:H7 in Biofilm on Food-Contact Surfaces by Sequential Treatments of Aqueous Chlorine Dioxide and Drying. Int. J. Food Microbiol..

[78] Han Q., Song X., Zhang Z., Fu J., Wang X., Malakar P.K., Liu H., Pan Y., Zhao Y. (2017). Removal of Foodborne Pathogen Biofilms by Acidic Electrolyzed Water. Front. Microbiol..

[79] Yu H., Liu Y., Li L., Guo Y., Xie Y., Cheng Y., Yao W. (2020). Ultrasound-Involved Emerging Strategies for Controlling Foodborne Microbial Biofilms. Trends Food Sci. Technol..

[80] Park S.Y., Jung S.-J., Ha S.-D. (2018). Synergistic Effects of Combined X-Ray and Aqueous Chlorine Dioxide Treatments against Salmonella Typhimurium Biofilm on Quail Egg Shells. LWT.

[81] Weng X., van Niekerk J., Neethirajan S., Warriner K. (2016). Characterization of Antimicrobial Efficacy of Photocatalytic Polymers against Food-Borne Biofilms. LWT—Food Sci. Technol..

[82] Chorianopoulos N.G., Tsoukleris D.S., Panagou E.Z., Falaras P., Nychas G.-J.E. (2011). Use of Titanium Dioxide (TiO_2_) Photocatalysts as Alternative Means for Listeria Monocytogenes Biofilm Disinfection in Food Processing. Food Microbiol..

[83] Barthomeuf M., Raymond P., Policarpo N., Castel X., Le Gendre L., Denis M., Pissavin C. (2017). Bactericidal Efficiency of UVA-Active Titanium Dioxide Thin Layers on Bacteria from Food Industry Environments. Mater. Technol..

[84] Jalvo B., Faraldos M., Bahamonde A., Rosal R. (2017). Antimicrobial and Antibiofilm Efficacy of Self-Cleaning Surfaces Functionalized by TiO2 Photocatalytic Nanoparticles against Staphylococcus Aureus and Pseudomonas Putida. J. Hazard. Mater..

[85] Pablos C., Govaert M., Angarano V., Smet C., Marugán J., Van Impe J.F.M. (2021). Photocatalytic Inactivation of Dual- and Mono-Species Biofilms by Immobilized TiO_2_. J. Photochem. Photobiol. B Biol..

[86] Shen H., Durkin D.P., Aiello A., Diba T., Lafleur J., Zara J.M., Shen Y., Shuai D. (2021). Photocatalytic Graphitic Carbon Nitride-Chitosan Composites for Pathogenic Biofilm Control under Visible Light Irradiation. J. Hazard. Mater..

[87] Manoharan R.K., Mahalingam S., Gangadaran P., Ahn Y.-H. (2020). Antibacterial and Photocatalytic Activities of 5-Nitroindole Capped Bimetal Nanoparticles against Multidrug Resistant Bacteria. Colloids Surf. B Biointerfaces.

[88] Nahar S., Jeong H.L., Kim Y., Ha A.J., Roy P.K., Park S.H., Ashrafudoulla M., Mizan M.F.R., Ha S.-D. (2021). Inhibitory Effects of Flavourzyme on Biofilm Formation, Quorum Sensing, and Virulence Genes of Foodborne Pathogens Salmonella Typhimurium and Escherichia Coli. Food Res. Int..

[89] KIM M.-J., LIM E.S., KIM J.-S. (2019). Enzymatic Inactivation of Pathogenic and Nonpathogenic Bacteria in Biofilms in Combination with Chlorine. J. Food Prot..

[90] Lim E.S., Koo O.K., Kim M.-J., Kim J.-S. (2019). Bio-Enzymes for Inhibition and Elimination of Escherichia Coli O157:H7 Biofilm and Their Synergistic Effect with Sodium Hypochlorite. Sci. Rep..

[91] Mazaheri T., Ripolles-Avila C., Hascoët A.S., Rodríguez-Jerez J.J. (2020). Effect of an Enzymatic Treatment on the Removal of Mature Listeria Monocytogenes Biofilms: A Quantitative and Qualitative Study. Food Control.

[92] Ripolles-Avila C., Ríos-Castillo A.G., Fontecha-Umaña F., Rodríguez-Jerez J.J. (2020). Removal of Salmonella Enterica Serovar Typhimurium and Cronobacter Sakazakii Biofilms from Food Contact Surfaces through Enzymatic Catalysis. J. Food Saf..

[93] Balabanova L., Podvolotskaya A., Slepchenko L., Eliseikina M., Noskova Y., Nedashkovskaya O., Son O., Tekutyeva L., Rasskazov V. (2017). Nucleolytic Enzymes from the Marine Bacterium Cobetia Amphilecti KMM 296 with Antibiofilm Activity and Biopreservative Effect on Meat Products. Food Control.

[94] Wang H., Wang H., Xing T., Wu N., Xu X., Zhou G. (2016). Removal of Salmonella Biofilm Formed under Meat Processing Environment by Surfactant in Combination with Bio-Enzyme. LWT—Food Sci. Technol..

[95] Vishwakarma V. (2020). Impact of Environmental Biofilms: Industrial Components and Its Remediation. J. Basic Microbiol..

[96] Lee H., Yoon Y. (2021). Etiological Agents Implicated in Foodborne Illness World Wide. Food Sci. Anim. Resour..

[97] USDA ERS—Economic Cost of Major Foodborne Illnesses Increased $2 Billion From 2013 to 2018. https://www.ers.usda.gov/amber-waves/2021/april/economic-cost-of-major-foodborne-illnesses-increased-2-billion-from-2013-to-2018/.

[98] Meroni G., Soares Filipe J.F., Drago L., Martino P.A. (2019). Investigation on Antibiotic-Resistance, Biofilm Formation and Virulence Factors in Multi Drug Resistant and Non Multi Drug Resistant Staphylococcus Pseudintermedius. Microorganisms.

[99] Giaouris E., Heir E., Hébraud M., Chorianopoulos N., Langsrud S., Møretrø T., Habimana O., Desvaux M., Renier S., Nychas G.-J. (2014). Attachment and Biofilm Formation by Foodborne Bacteria in Meat Processing Environments: Causes, Implications, Role of Bacterial Interactions and Control by Alternative Novel Methods. Meat Sci..

[100] Shivaprasad D., Taneja N.K., Lakra A., Sachdev D. (2021). In Vitro and in Situ Abrogation of Biofilm Formation in E. Coli by Vitamin C through ROS Generation, Disruption of Quorum Sensing and Exopolysaccharide Production. Food Chem..

